# Do Gendered Psychosocial Factors Explain Sex Differences in White Matter Hyperintensities?

**DOI:** 10.1093/geroni/igaa057.2693

**Published:** 2020-12-16

**Authors:** C Elizabeth Shaaban, Caterina Rosano, Heather Shattuck-Heidorn, Sara Godina, Minjie Wu, Andrea Rosso

**Affiliations:** 1 University of Pittsburgh, Pittsburgh, Pennsylvania, United States; 2 Women and Gender Studies Program, University of Southern Maine, Portland, Maine, United States; 3 University of Pittsburgh Graduate School of Public Health, Pittsburgh, Pennsylvania, United States; 4 School of Public Health, University of Pittsburgh, Pittsburgh, Pennsylvania, United States

## Abstract

Women have a greater burden than men of white matter hyperintensities (WMH), a marker of cerebral small vessel disease (cSVD). Psychosocial factors including education, household income, neighborhood socioeconomic status (nSES), happiness, and depression may differ by gender and could explain women’s higher burden of WMH. In a cohort of older adults (N=250, median age=82, 58% women, 39% Black), we found that women had lower education, household income, nSES and were less happy and more depressed. Race stratified Spearman correlations showed women had greater whole brain WMH volume in white participants only (white: rho=0.23, p=0.004; Black: rho=-0.05, p=0.64). In partial Spearman correlations, education, happiness, and depression attenuated but did not fully explain the relationship when added individually or all together to the model for whites (fully adjusted rho=0.19, p=0.03). Gendered psychosocial factors may partially explain sex differences in WMH; interventions targeting these factors may reduce cSVD burden, particularly in white women.

